# Rapid component of excess post-exercise oxygen consumption of children of different weight status after playing active video games

**DOI:** 10.1186/s12887-021-02528-z

**Published:** 2021-02-15

**Authors:** Caio Victor Sousa, Jungyun Hwang, Herbert Gustavo Simoes, Kyung Jin Sun, Amy Shirong Lu

**Affiliations:** 1 Health Technology Lab, College of Arts, Media & Design; Bouvé College of Health Sciences, Northeastern University, Boston, MA USA; 2 Department of Aging and Geriatric Research, University of Florida, Gainesville, FL USA; 3 Graduate Program in Physical Education, Catholic University of Brasilia, Brasilia, Brazil

**Keywords:** Exergame_1_, Active video game_2_, EPOC_3_, Oxygen uptake_4_, Children_5_, Kinect_6_, Xbox_7_

## Abstract

**Background:**

Excess post-exercise oxygen consumption (EPOC) of children could indicate the potential of an exercise therapy to treat or prevent obesity. However, EPOC as a result of playing active video games (AVG) has been poorly investigated. Therefore, we aimed to investigate the rapid component of EPOC of children with healthy weight and overweight/obesity (according to their BMI percentile) after playing AVGs that feature predominately upper body (UB) and whole-body (WB) movement.

**Methods:**

Twenty-one children with healthy weight (BMI percentile < 85%) and with overweight/obesity (BMI percentile ≥ 85%) randomly underwent two 10-min AVG sessions (UB and WB). The heart rate (HR), minute ventilation (VE), oxygen consumption (VO_2_) and carbon dioxide production (VCO_2_) were recorded during exercise and post-exercise recovery period. For the rapid component of EPOC in each AVG session, measurements were recorded every 15 s for 5-min of post-exercise recovery. The rate of perceived exertion (RPE) was also measured immediately before and after each AVG play.

**Results:**

Children with overweight/obesity had a higher average of absolute VE, VO_2_, and VCO_2_ than their healthy-weight counterparts (BMI percentile < 85%; *n* = 21) during post-exercise recovery. RPE, HR, and HR% were not different between the game sessions and weight groups. Children with overweight/obesity showed a higher absolute VO_2_ during EPOC than healthy-weight children in both game sessions, but relative VO_2_ was higher in healthy-weight children during EPOC. No differences were observed for EPOC between UB and WB sessions.

**Conclusions:**

Children with overweight/obesity had a greater EPOC than healthy-weight children after AVG sessions in terms of absolute oxygen values, whereas healthy-weight children have higher EPOC considering relative VO_2_ when controlling for body mass. UB and WB AVGs induced a similar EPOC among children with healthy weight and overweight/obesity. As UB and WB AVGs induce the rapid component of EPOC in children regardless their weight status, AVGs could be used as an exercise method to treat and prevent child obesity.

## Introduction

In the United States, 18.5% of children and adolescents are obese [1]. Children with obesity and overweight frequently have elevated blood pressure and abnormal fasting glucose levels [2]. Moreover, children with obesity are more likely to become obese young adults [3].

One of the most popular strategies used to treat or prevent obesity is the reduction of sedentary behavior and increase physical activity levels [4]. In the last decade, U.S. children have tripled the time spent in sedentary video games [5, 6]. A recent study has found that children aged 8 to 12 play video games for around 1.6 h per day [6]. However, active video games (AVGs) can elicit moderate-to-vigorous physical activity in both adults [7] and children [4, 8]. AVGs are video games that require more physical activity than the conventional hand-controlled games [9]. AVGs can prompt similar physiological demands as other (non-media) activities, such as dancing and cycling [10, 11]. AVGs have therefore emerged as an alternative for increasing movement and physical activity in children [10].

The literature on new AVG technologies displays a considerable heterogeneity in games, which is likely due to new releases of cutting-edge technologies every year [12]. For instance, one of the earlier AVGs was Dance Dance Revolution [13], which requires the player to move their feet across four small squares on a one-square-meter dance pad platform to reproduce the dancing moves of an avatar on screen. Nintendo Wii later added an innovative introduction in the form of a wireless remote controller with a movement sensor that enables the player to use the wireless controller as a game instrument to play virtual sports (i.e. tennis, boxing). However, the player still required the controller to record their movements in the game being played [14]. More recently, Microsoft has added the ability to sense every movement of the human body and to reproduce the user’s body movements in an onscreen virtual avatar through the Kinect sensor for Xbox games [15]. This technology has enabled many possible AVG innovations and facilitated existing features for dancing games as well as virtual sports and fitness games [15].

The heterogeneity of AVGs presents an advantage of this method, which allows people with a diverse range of mobility to play/exercise. People with extremity disabilities can play AVGs targeting the upper-body (UB) or the lower-body (LB) based on their particular limitations. Other people who need to avoid the risk of acute peripheral fatigue or muscular imbalances in the long term can play whole-body (WB) AVGs, which involve more body segments [10]. Researchers have demonstrated that WB games elicit a higher energy expenditure than UB or LB, likely due to the greater amount of muscle mass involved [8].

While energy expenditure during an activity, such as an AVG, give us information regarding the possible impact that play could have on preventing or treating obesity, measures during the post-exercise recovery period also presents an opportunity to investigate caloric expenditure and substrate oxidation rate. After exercise, the body shifts from using carbohydrate (CHO) to fat as its main fuel substrate [16]. This change in substrate utilization reflects the corresponding observation that oxygen consumption remains elevated after exercise, a condition called excess postexercise oxygen consumption (EPOC) [17]. An increased EPOC is particularly relevant as it may indicate a higher utilization of fat due to the greater oxygen cost of fat utilization (compared to CHO utilization). Thus, two different activities can have the same energy cost while producing a different EPOC. The activity with a greater EPOC uses more fat as an energy source after exercise and thus is a more efficient method for treating or preventing obesity [17].

EPOC is influenced by many factors, including fitness level, nutritional status, physical activity, and level of adiposity [16, 18, 19]. Examining substrate use during the recovery phase would help to clarify the role of excess body fat or type of exercise on fat oxidation. However, to the best of our knowledge, there have been no studies investigating the EPOC of children playing AVGs, let alone the potential roles of body fat or the type of game. Such information would show whether a certain type of AVG would elicit higher EPOC and therefore is more effective for obesity prevention and intervention. Besides, tailored exercise therapies using AVG could be more effective for children with overweight/obesity if their EPOC response is different from healthy-weight children.

Therefore, we aimed to investigate the rapid component of oxygen consumption and substrate utilization of children with healthy weight and overweight/obesity after playing UB and WB AVGs. Our initial hypotheses were that WB AVG would elicit a greater EPOC than UB AVG, and that overweight/obese children would produce a greater absolute EPOC than children with healthy weight regardless of the type of game.

## Methods

### Design and ethical procedures

Our cross-sectional study was approved by the institutional review board at the Northeastern University. All children participants signed a written informed assent and their parents or guardians, a consent for the children’s participation. All protocols were carried out in accordance with relevant guidelines and regulations.

### Participants

We recruited participants from a community in the Greater Boston area through web advertisements and flyers. We included boys and girls from all ethnic backgrounds. Our inclusion criteria were: aged between 8 to 12 years; free of attentional disorders or physical disability; free of any medications that could affect the central nervous system; had not previously played the AVG we intended to use in our study. Of our initial pool of 421 survey respondents, 82 children were eligible; from the latter group, we recruited 42 children. We excluded 10 participants from our final analysis due to invalid metabolic data (e.g., mask leakage; the participant asked to drink water during recovery). We have reported the details of our selection process in a previous paper [8].

### General procedures

All study sessions were performed on weekend mornings between 8 AM and 12 PM. Participants and their parents came to the lab for a single 90-min visit. They were instructed not to eat or drink anything other than water for at least 2 h before the study. Participants were also asked to refrain from strenuous exercise and caffeinated beverages for 24 h before the visit. At the start of their visit, they received orientation regarding the study procedures and signed the informed assent/consent. They then completed a Physical Activity Questionnaire for Older Children (PAQ-C) [20] and had their anthropometric measurements taken. PAQ-C is correlated with cardiorespiratory fitness. The average PAQ-C score ranges between 3.1 to 3.2 for boys, and 2.8 to 3.0 for girls [21]. After that, the participant had the devices affixed to them and completed the AVG sessions.

### Anthropometry

Height was measured using a 0.1 cm scale (ShorrBoard, Weight and Measure, LLC, Olney, MD), and weight was measured using a SECA scale (SECA Inc., Chino, CA), both according to the protocol previously described [8]. Body mass index (BMI) was calculated (kg/m^2^), and BMI percentile was produced for use with the Centers for Disease Control and Prevention (CDC) growth charts [22]. Participants were then identified, based on their BMI percentile, as with healthy weight (<85th percentile) or with overweight/obesity (≥85th percentile).

### Oxygen uptake, heart rate and rate of perceived exertion

The metabolic analyzer, a COSMED K4b^2^ (COSMED, Rome, Italy), was calibrated following the manufacturer’s instructions and tested to ensure no leakage of air. The face mask (Hans Rudolph Inc., Kansas City, MO) was fitted to the participant after 20-min seated at rest. The 5-min resting metabolic variables were measured immediately after the resting period. During resting, AVG sessions, and post-exercise stages, metabolic variables were assessed using the breath-by-breath method with indirect calorimetry through the metabolic analyzer. During the 5-min recovery the participants were asked to remain seated with their masks. The average of each 15-s intervals was downloaded. The Energy expenditure (EE) was expressed in kcal/min, and minute ventilation (VE), oxygen uptake (VO_2_) and carbon dioxide output (VCO_2_) were expressed in milliliters per minute (ml/min). We also calculated the relative VO_2_ in ml/kg/min, and respiratory exchange ratio (RER). As previously stated, 10 participants were removed from the analysis because they were not able to keep at rest with mask after exercising, generating a great variability in respiratory data.

A Polar H7 Bluetooth heart rate (HR) sensor was placed on the chest with a soft strap to measure HR continuously during the AVG sessions. The HR average of each 10-s intervals was downloaded, and values were expressed in beats per minute (bpm). Participants provided their rate of perceived exertion (RPE) immediately after each AVG session using the children’s OMNI scale (category range 1–10: 0 = extremely easy; 10 = extremely hard) [23].

### Active video game sessions

We selected two AVGs we have used previously to induce minutes of moderate-to-vigorous physical activity (MVPA) [8], based on the movement pattern and intensity. Both games were played using a Kinect camera monitor sensor connected to an Xbox One console (Microsoft Inc., Redmond, WA). The games were *Fruit Ninja* [24], in which the player needs to slice different virtual objects using the arms (thus comprises predominantly upper-body movements), and *Kung-Fu for Kinect* [25], in which the player needs to kick and punch virtual enemies (thus involved whole-body movements). Participants played both games in a randomized order with a 5-min interval between games. Each AVG playing session lasted for 10 min. Each game’s difficulty level was set to “medium” to ensure the comparability of the exercise intensity. We have also employed the RPE and average HR as measures of internal load to ensure that the exercise intensity did not differ between the groups.

### Statistical analysis

Data were tested for normality and homogeneity using Kolmogorov-Smirnov and Levene’s tests, respectively. Means and SDs were calculated and reported for all dependent variables. Age and body composition parameters were compared using independent t-tests. General linear model (GLM: two-way ANOVA) with Group (healthy weight vs. overweight/obese) and Game (UB vs. WB AVG) as random factors was conducted for the metabolic variables, HR, and RPE. When significant effects were identified, we used pairwise comparisons (least significant difference, LSD) for each factor to identify specific group differences. The EPOC data (VO_2_) were transformed into net VO2 (subtracting resting values individually) and both, raw and net VO_2_ were fitted into an exponential function using the least-square technique. Then the constant plateaus, with their respective confidence intervals (CI: 95%), were compared between conditions. The area under the curve (AUC) during EPOC was calculated individually for each AVG game. Each participant’s AUCs were then compared with the GLM as the other variables. Power calculations were conducted post hoc using G*Power and all other statistical procedures were conducted using Statistical Software for the Social Sciences (SPSS v26.0; IBM, Armonk, NY). Power calculations were then conducted using the actual sample size (*n* = 32) for the main analysis with a two-way 2 × 2 ANOVA design; our calculations indicated sufficient power to detect moderate effect sizes (d = 0.6), and our analysis was assured to have a power of 89.7%. The significance level was set at *p* < 0.05.

## Results

The healthy and overweight/obese groups differed significantly in height (*p* = 0.003) and as expected, in their weight (*p* < 0.001), BMI (*p* = 0.001) and BMI percentile (*p* = 0.001). Additionally, participants also differed in their resting VE (*p* = 0.010), absolute VO_2_ (*p* = 0.001), and VCO_2_ (*p* = 0.004). No differences were identified for self-reported PA levels (*p* = 0.756). See Table 1. No significant Game or Group effect were identified for both exercise intensity measures, RPE and average HR (Table 2**)**.

**Table 1:** Age, body composition parameters, and resting respiratory values. Data expressed as mean and standard deviation (±)

		Healthy weight (*n* = 21)	Overweight (*n* = 11)
Sex (n, %)	Girls	6 (28.6%)	4 (36.4%)
	Boys	15 (71.4%)	7 (63.6%)
Ethnics (n, %)	Asian	0 (0%)	0 (0%)
	White or Caucasian	12 (57.1%)	5 (45.5%)
	Black or African American	4 (19.1%)	5 (45.5%)
	American Indian or Alaska Native	1 (4.8%)	0 (0%)
	Native Hawaiian or Pacific Islander	4 (19.1%)	0 (0%)
	Other	1 (4.8%)	1 (9.0%)
Age (years)		9.6 ± 1.4	10.4 ± 1.4
Height (cm)		142.2 ± 12.8	152.7 ± 9.8 *
Weight (kg)		34.5 ± 9.5	60.0 ± 18.7 *
BMI (kg/m^2^)		16.8 ± 2.8	25.2 ± 5.2 *
BMI percentile		41.1 ± 28.9	94.8 ± 3.9 *
PAQ-C Score		2.66 ± 0.56	2.72 ± 0.42
VE (l/min)		8.4 ± 1.8	11.4 ± 3.3 *
VO_2_ (ml/min)		293.4 ± 56.1	385.3 ± 71.8 *
VCO_2_ (ml/min)		261.4 ± 56.4	340.5 ± 72.4 *
VO_2_ (ml/kg/min)		8.5 ± 3.2	7.5 ± 1.2
RER		0.92 ± 0.1	0.92 ± 0.1

*BMI* Body mass index, *PAQ-C* Physical activity questionnaire for older children, *VE* Minute ventilation, *VCO*_*2*_ Carbon dioxide output, *VO*_*2*_ Oxygen uptake, *RER* Respiratory exchange ratio; *: significantly different (*p* < 0.05) from healthy weight group

**Table 2:** Measured exercise intensity of UB and WB AVGs by weight status. Data expressed as mean and standard deviation (+)

	Healthy weight *(n* = 21)		Overweight/obesity (*n* = 11)	
	UB	WB	UB	WB
RPE (1–10 scale)	3.8 ± 2.3	4.1 ± 2.3	4.4 ± 2.0	4.7 ± 2.1
Average HR (bpm)	98.1 ± 12.7	96.4 ± 15.8	97.6 ± 13.4	95.7 ± 14.8
Average HR%	47.6 ± 6.2	47.4 ± 7.1	48.4 ± 6.4	47.5 ± 7.2

*UB* Upper-body, *WB* Whole-body, *RPE* Rating of perceived exertion, *HR* Heart rate, *HR%* Percentage of estimated maximal heart rate

**Fig. 1:**
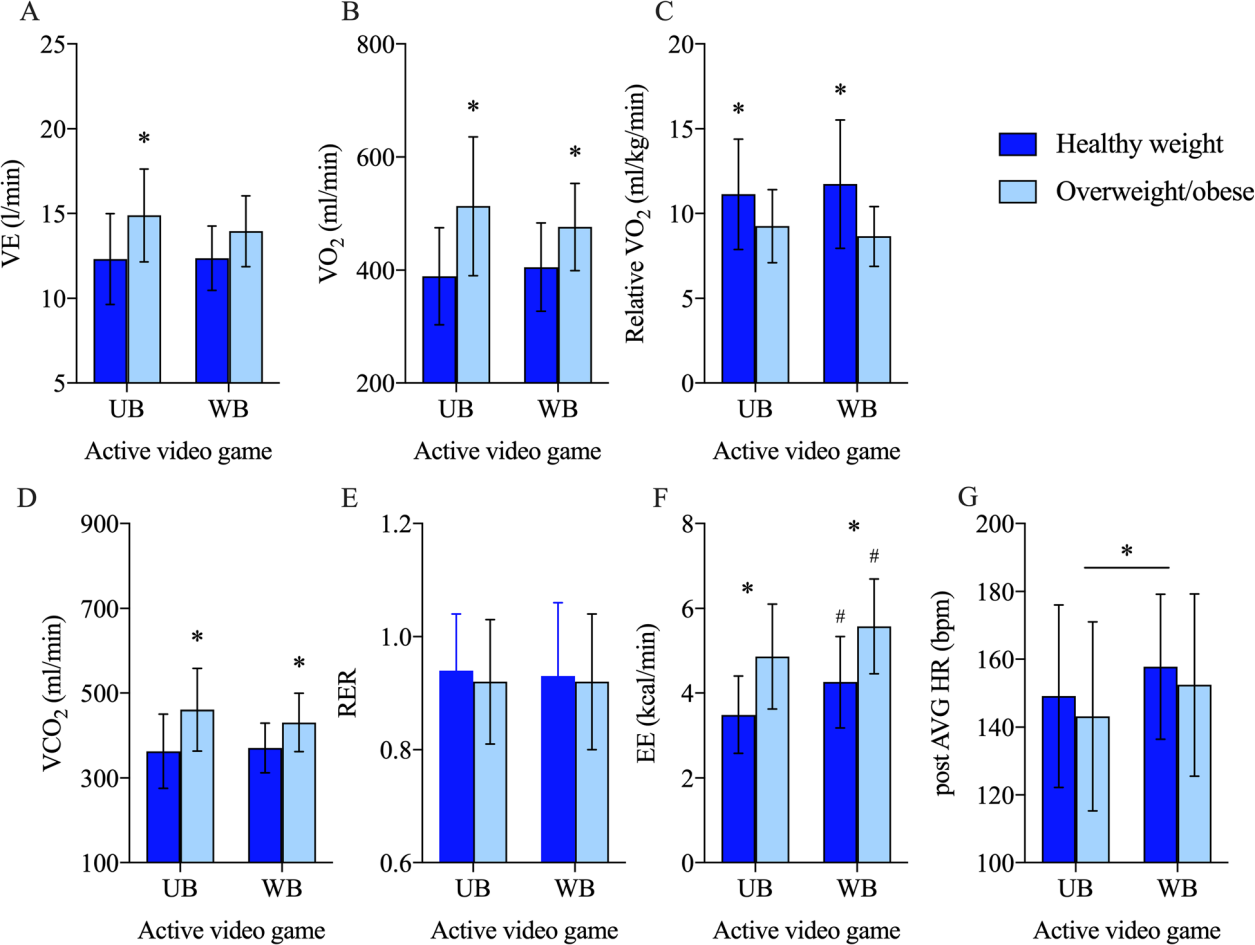
Metabolic response of children with healthy weight and overweight playing UB (upper body game: Fruit Ninja®) and WB (whole-body game: Kung Fu Kinect®) active video games; *: difference (*p* < 0.05) between weight groups; ^#^: marginal difference (*ps* ≤ 0.086) between weight groups

Two-way ANOVAs of data relative to immediate post-game analysis indicated a group effect (healthy weight vs. overweight/obese) for minute ventilation (F = 6.5; *p* = 0.018; Fig. 1a), absolute VO_2_ (F = 9.9; *p* = 0.004; Fig. 1b), relative VO_2_ (F = 5.2; *p* = 0.032; Fig. 1c), and VCO_2_ (F = 8.2; *p* = 0.008; Fig. 1d). No significant effects were identified for RER (Fig. 1e). Pairwise comparisons showed that the group with overweight/obesity had higher values of VE, absolute VO_2_, VCO_2_ and lower relative VO_2_ than healthy-weight group after the UB AVG playing session (Fig. 1). Energy expenditure showed significant group- (F = 17.7; *p* < 0.01) and game-effect (F = 30.2; *p* < 0.01). Pairwise comparisons indicated higher EE for WB in both groups, and higher EE for children with overweight/obesity in both games (Fig. 1f), whereas, post-playing HR had a significant game-effect (F = 4.7; *p* = 0.037), see (Fig. 1g).

**Fig. 2:**
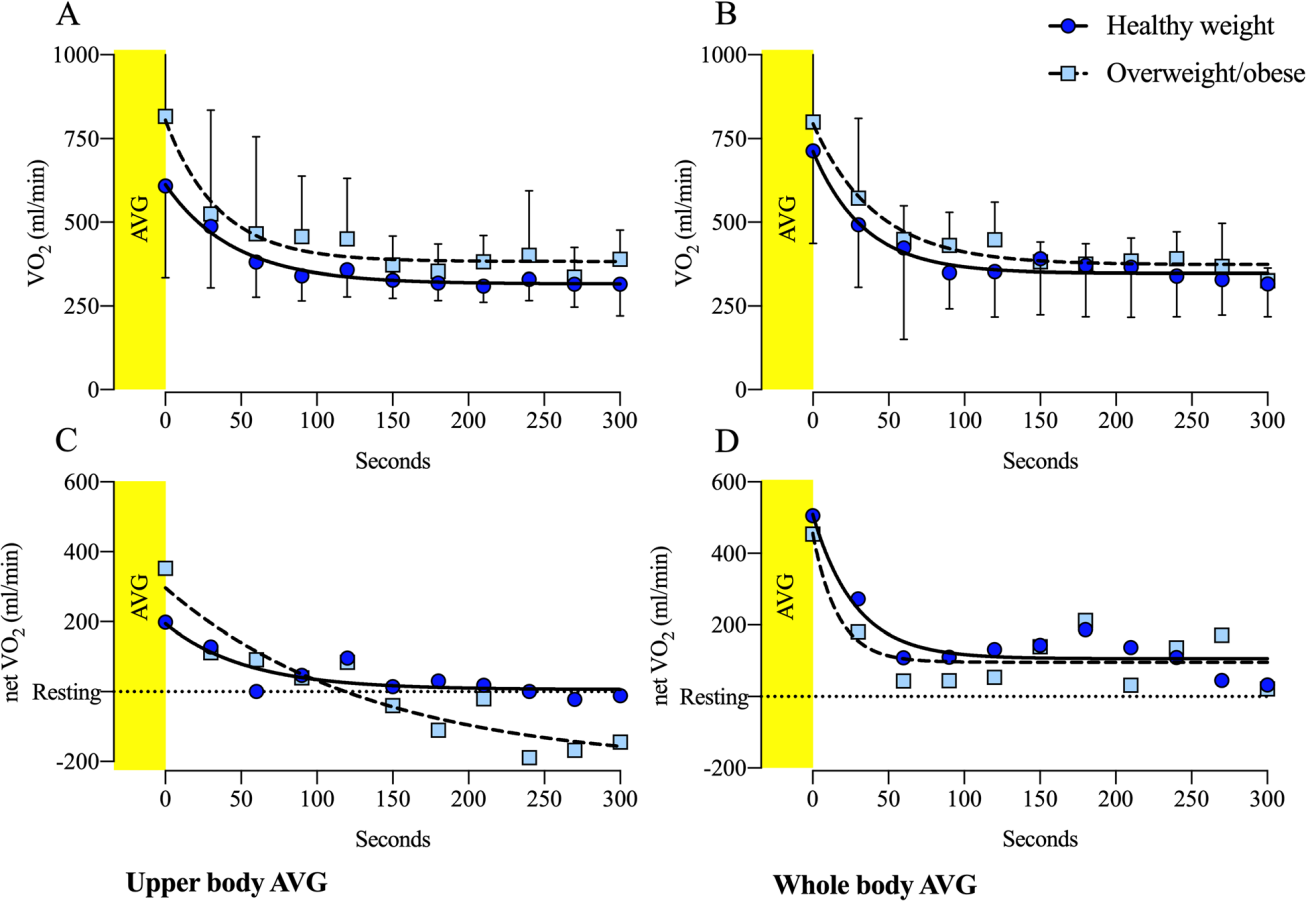
Raw oxygen uptake (**a** and **b**) and net oxygen uptake (**c** and **d**) response over time with regression analysis of EPOC of healthy weight and children with overweight/obesity after playing UB and WB AVGs

The EPOC data were analyzed by an exponential function for each group and condition for the immediate 5 min following each game session. All functions showed a good fit with the average EPOC data from each group: WB: healthy weight (R^2^ = 0.9661), overweight/obese (R^2^ = 0.9663); UB: healthy weight (R^2^ = 0.9826), overweight/obese (R^2^ = 0.9443). Additionally, children with overweight/obesity showed a higher plateau than healthy-weight children in both WB [373 (95% CI: 342–400) vs. 347 (95% CI: 324–369)], and UB [383 (95% CI: 344–416) vs. 316 (95% CI: 301–330)] game session. See Fig. 2a and Fig. 2b. Considering net VO_2_ values, children with overweight/obesity showed a declining oxygen uptake, ending the with an average value below resting VO_2_ after UB (Fig. 2c). For WB, net VO2 showed similar plateau between the weight groups (Fig. 2d).

## Discussion

We aimed to investigate the rapid component of EPOC in children with healthy weight and overweight/obesity after playing UB and WB AVGs, considering the following hypotheses: 1) WB AVG would elicit a higher EPOC than UB AVG; 2) children with overweight/obesity have a higher EPOC than healthy-weight children regardless of the type of game. Our main findings did not support our first hypothesis as there was no significant difference between the two types of AVGs. Our second hypothesis was supported: children with overweight/obesity showed a greater oxygen consumption than healthy-weight children after both WB and UB AVG sessions. However, after controlling oxygen uptake by body weight, the findings were reversed: EPOC was higher in healthy-weight children. Additionally, we did not find differences between the two game types concerning EPOC in healthy weight and children with overweight/obesity.

We are the first to investigate the effect of the type of AVG over EPOC. When previous researchers compared different exercise modes, although WB or LB exercises require the involvement of more muscle mass [26], there is no difference in EPOC responses for traditional exercise types, which corroborates our results obtained using AVGs. For instance, the results of a two-arm cross-sectional study comparing arm crank exercise versus cycling indicated that there is no EPOC difference between these exercise modes [27]. A comparison of UB exercise (arm crank) at two different exercise intensities (35 and 70% of VO_2peak_) suggested an EPOC pattern similar to conventional cycling in the ergometer (lower body only), with a greater EPOC magnitude at higher intensities [28].

Although the type of game did not affect EPOC, we did find differences between children with healthy weight and overweight/obesity, which corroborated a previous study on 10 children with obesity and 10 non-obese controls using a running/walking exercise protocol that only the obesity group showed higher RER values after exercise [29]. Conversely, a comparison of adults with vs. without obesity indicated a higher EPOC in lean men as well as higher concentrations of growth hormone and cortisol in the obese group [16]. These results suggest that hormones play an important role during EPOC, potentially as a part of substrate utilization. During EPOC, a shift from CHO use towards greater fat use is expected, reflected by a drop in RER values [17]. Previous researchers demonstrated that this decrease is greater in obese individuals and suggested that it may be due to reduced insulin sensitivity, which in turn reduces glucose uptake and increases the need to use fat as an energy substrate [16, 30]. However, we did not find a difference in RER values between children with healthy weight and overweight/obesity. That may be because insulin resistance is less common in children with obesity than in adults with obesity [31].

The hours after exercise represent the slow component of EPOC. Since we could not assess this part of EPOC, this may represent a limitation on our results. However, previous AVG research indicates that the exercise intensities necessary to elicit prolonged EPOC are unlikely to be tolerated by non-athletic individuals [32] and possibly are not well tolerated by children. Our participants also could only play the AVGs for a limited time during a single visit, which may constitute another limitation. The fact that the two game sessions were performed consecutively is a limitation of the design, since first task may affect the following. Nonetheless, we randomized the game order to minimize this issue. Finally, we used multiple proximate measures and controls, such as game difficulty level, RPE and average HR, to equalize exercise intensity between groups instead of assessing a relative percentage of maximal oxygen uptake (VO_2_max). Further investigation is needed to identify methods to equalize exercise intensity during active video games. From a practical perspective, AVGs could be used as a strategy to increase the physical activity necessary to elicit EPOC regardless of the game type (UB or WB) in both groups, with and without overweight/obesity. AVGs may thus be more efficient in producing weight loss in children with overweight/obesity.

## Conclusions

Both UB and WB AVG induce the rapid component of EPOC in children with healthy weight and overweight/obesity. Additionally, children with overweight/obesity showed a greater EPOC than healthy-weight children after AVG sessions when considering absolute oxygen values. When oxygen uptake is relative to their body weight, healthy-weight children showed a higher EPOC than children with overweight or obesity. Our results show the potential of AVGs to elicit higher fuel utilization at rest after a play session, offering potential for child obesity prevention and intervention. Future researchers could investigate the slow component of EPOC after AVG and factors related to the potential mechanisms of the change in substrate utilization during EPOC, such as insulin resistance.

## Data Availability

The datasets used and/or analyzed during the current study are available from the corresponding author on reasonable request.
